# Hypermetabolism in B–lymphocytes from malignant hyperthermia susceptible individuals

**DOI:** 10.1038/srep33372

**Published:** 2016-09-20

**Authors:** Kerstin Hoppe, Guido Hack, Frank Lehmann–Horn, Karin Jurkat–Rott, Scott Wearing, Alberto Zullo, Antonella Carsana, Werner Klingler

**Affiliations:** 1 Department of Anaesthesia, Intensive Care Medicine and Pain Therapy, Frankfurt University, Theodor–Stern–Kai 7, 60590 Frankfurt, Germany; 2 Division of Neurophysiology in the Center of Rare Diseases, Ulm University, Albert–Einstein–Allee 23, 89081 Ulm, Germany; 3 Institute of Health and Biomedical Innovation, Queensland University of Technology, 60 Musk Avenue, Kelvin Grove 4059, Australia; 4 Department of Science and Technologies, University of Sannio, Benevento, Italy and CEINGE Advanced Biotechnologies s.c.ar.l, Naples, Italy; 5 Department of Molecular Medicine and Medical Biotechnology, University of Naples Federico II, Naples, Italy; 6 Department of Neuroanaesthesiology, Neurosurgical University, Ludwig–Heilmeyerstr. 2, 89312 Guenzburg, Germany

## Abstract

Malignant hyperthermia (MH) is a pharmacogenetic disorder of skeletal muscle metabolism which is characterized by generalized muscle rigidity, increased body temperature, rhabdomyolysis, and severe metabolic acidosis. The underlying mechanism of MH involves excessive Ca^2+^ release in myotubes via the ryanodine receptor type 1 (RyR1). As RyR1 is also expressed in B–lymphocytes, this study investigated whether cellular metabolism of native B–lymphocytes was also altered in MH susceptible (MHS) individuals. A potent activator of RyR1, 4–chloro–m–cresol (4-CmC) was used to challenge native B-lymphocytes in a real–time, metabolic assay based on a pH–sensitive silicon biosensor chip. At the cellular level, a dose–dependent, phasic acidification occurred with 4–CmC. The acidification rate, an indicator of metabolic activation, was significantly higher in B–lymphocytes from MHS patients and required 3 to 5 fold lower concentrations of 4–CmC to evoke similar acidification rates to MHN. Native B–lymphocytes from MHS individuals are more sensitive to 4–CmC than those from MHN, reflecting a greater Ca^2+^ turnover. The acidification response, however, was less pronounced than in muscle cells, presumably reflecting the lower expression of RyR1 in B–lymphocytes.

In skeletal muscle, Ca^2+^ is released from the sarcoplasmic reticulum via the ryanodine receptor protein (RyR1) and mediates crossbridge cycling of contractile filaments. Activation of myosin ATP–ase requires energy, which is replenished by glycolysis and cellular respiration, the latter of which leads to accumulation of acidic metabolites. Volatile anesthetics are potent activators of abnormal RyR1. In genetically predisposed individuals, these drugs can trigger life–threating hypermetabolic events known as malignant hyperthermia (MH). In such cases, undue activation of muscle metabolism causes muscle rigidity, hyperthermia, and severe acidosis^1^,^2^,^3^. Given the high mortality rate associated with the syndrome, it is particularly important to identify MH susceptibility.

Diagnosis of MH is currently based on the *in vitro* contracture test (IVCT) used by the European malignant hyperthermia group or the caffeine halothane contracture test (CHCT) used by the North American malignant hyperthermia group, in which surgically excised muscle develops contractures on exposure to select chemicals as an indirect marker of myoplasmic Ca^2+^–concentration^4^. In many cells, other than skeletal muscle, Ca^2+^ plays an important role as a secondary messenger and activator of cellular processes and its intracellular concentration is regulated strictly in the range of 10^−9^ to 10^−3^ molar. RyR1 is expressed in the membranes of internal Ca^2+^ stores of B–lymphocytes, where cell proliferation, gene expression, antibody secretion, and cytokine production are influenced by Ca^2+^ levels^5^,^6^. Indeed, cultured or immortalized B–lymphocytes from MH susceptible (MHS) patients have been shown to have a heightened sensitivity to RyR1 activators compared to MH-negative patients (MHN) and to exhibit an increased production of the endogenous pyrogen IL–1β^7^,^8^.

Previously, we have shown with the use of a highly sensitive proton biosensor assay, that MHS can be identified by measuring the cellular acidification rate of both cultivated myotubes and EBV-immortalized lymphoblastoid cell lines^9^,^10^. Nevertheless, muscle biopsy is an invasive procedure and the immortalization of lymphoblastoid cells is a laborious and time consuming method. Therefore, we investigated whether evaluating metabolic activity in native B–lymphocytes might offer a future minimally–invasive approach for MH–diagnostics. Here we show that agarose trapped native B–lymphocytes display increased cellular acidification in MHS compared to MHN.

## Materials and Methods

### Patients

A consecutive sample of 23 patients referred to the MH–center (Ulm, Germany) over a 2–year period was recruited for the study. Indications for MH testing were; (1) an adverse anesthetic event of the patient or a close relative, (2) a family–history of established MH, or (3) chronic isolated creatine kinase elevation. Four individuals without suspicion of neuromuscular disease (e.g. MH) served as healthy controls. Participant numbers were determined based on previously published acidification rates measured using a similar method in myotubules^9^. Assuming equal group variance, the projected sample was estimated to have sufficient power (β = 0.80) to detect an 30% difference in acidification rates between groups at an α level of 0.05.

Ethics approval for this study was obtained from the Ethics Committee of University of Ulm, Germany. Informed consent was obtained from patients prior to their participation. All methods were carried out in accordance with the approved guidelines and signed informed written consent was collected from all participants prior to participation in the study.

Gene mutation screening was performed on all MHS individuals as described in detail previously^10^,^11^. Ethylendiamintetraacetate blood samples of fifteen MHS patients were genetically screened for mutations in all 106 exons of the RyR1 gene and additionally for known mutations of CACNA1S. Blood cells were then haemolysed and DNA extracted and amplified by polymerase chain reaction (PCR) for further analysis. PCR samples were mixed with the wild–type amplicons, denatured at 95 °C for 5 min and then cooled at room temperature to allow heteroduplexes to form. Amplicons with an altered DHPLC elution profile compared to wild–type amplicons were directly sequenced with dye–terminator chemistry (Applied Biosystems).

### Chemicals and Solutions

The following chemicals and solutions were used in experimental measurements. Chemicals were ordered from Sigma–Aldrich, Steinheim, Germany, unless stated otherwise. Concentrations have been expressed in mM unless noted otherwise:

#### Krebs–Ringer solution

NaCl 118, KCl 3.4, MgSO_4_ 0.8, KH_2_PO_4_ 1.2, glucose 11.1, NaHCO_3_ 25.0, CaCl_2_ 2.5, pH 7.4.

#### Density gradient centrifugation for leukocyte isolation

Ficoll with 1.077 g/ml density (L6115, Biochrom, Berlin, Germany), phosphate buffer solution without Ca^2+^ and Mg^2+^ (L1825, Biochrom, Berlin, Germany)^12^.

#### Cryopreservation

Hanks medium (L2015, Biochrom, Berlin, Germany) with 20% fetal calf serum (F2442, Sigma, USA)^13^.

#### Thawing of leukocytes

Hanks medium (L2015, Biochrom, Berlin, Germany) with 10% fetal calf serum at 37 °C, buffered Roswell Park Memorial Institute medium with 26.7 ml/L NaHCO_3_ (R6504, Sigma, Deisenhofen, Germany) and 1% fetal calf serum^14^.

#### Isolation solution

Phosphate–buffered saline with and without Ca^2+^ and Mg^2+^ supplemented with 0.1% bovine serum albumin (BSA) (A2153, Sigma, Deisenhofen, Germany), unbuffered RPMI medium (4.78 ml/L 5 M NaCl, osmolarity 280 mOsmol) (R6604, Sigma Deisenhofen, Germany) with 1% fetal calf serum, negative isolation kit, (113.13 Dynal, Norway).

#### Cytosensor test solution

Unbuffered RPMI medium (R6504, Sigma, Deisenhofen, Germany) with 1% fetal calf serum, low melt agarose (1.5%) (F50082, FMC, USA) resolved in unbuffered RPMI medium.

Stock solutions of the trigger reagents caffeine and 4–chloro–m–cresol (4–CmC) were prepared in bi–distilled water. Halothane was applied using a vaporizer (Vapor 19.1, Draeger, Lübeck, Germany). Caffeine was purchased from Merck (Darmstadt, Germany), 4–CmC from Fluka (Neu–Ulm, Germany) and halothane from Zeneca (Plankstadt, Germany).

### IVCT

In accordance with the recommendations of the European Malignant Hyperthermia Group, MHS was diagnosed using the *in vitro* contracture test as described in detail elsewhere^4^,^15^. In brief, this test determines the contractile sensitivity of surgically excised muscle specimens to halothane and caffeine. Biopsies were taken from the vastus lateralis muscle under regional anesthesia. Basal tension and the twitch response to supramaximal electrical stimulation (30 to 70 V, 0.2 Hz, 1 ms) was recorded with a force transducer. Muscle bundles from MHS patients have lower contractile thresholds to caffeine and halothane than MHN. A positive result for MHS was defined as a contracture force of ≥2 mN at a caffeine concentration ≤2 mM and a halothane concentration ≤2% v/v^4^. Effects of 4–CmC (concentration range, 25–200 μM) were also tested on the excised muscles biopsies. In addition, biopsies were sent for standard histological examination of core formation, polygonal shape of myofibers, nuclear location, fiber type composition and the presence of inflammatory cell infiltrate.

### Isolation of B–lymphocytes

Blood samples (18 ml) from patients referred for an *in vitro* contracture test were also taken for isolation of B–lymphocytes. After Ficoll separation, the remaining leucocytes were temporarily stored by cryopreservation at −70 °C. An indirect isolation method was then employed to avoid activation of cellular metabolism by direct binding of CD19–antibodies to B–lymphocytes^16^,^17^. Leukocytes were incubated with a mixture of primary antibodies against CD2, CD7, CD3, CD14, CD16, and CD56 for 20 minutes at 4 °C. In a second step, secondary monoclonal antibodies with magnetic beads were conjugated^18^,^19^. With application of a stationary magnetic field, the B–lymphocytes remained in the supernatant. The cells were then suspended in agarose for the acidification measurements and stimulated with increasing doses of 4–CmC, a potent RyR1 agonist.

### Mononuclear cells and EBV-transformed cell lines

Whole blood was collected in EDTA-treated tubes and mononuclear cells were isolated by Ficoll-Hypapue density gradient centrifugation. For infection with Epstein-Barr virus (EBV), mononuclear cells were exposed to supernatants of the B95.8 cell line according to standard procedures^20^. Cells were cultured in Iscove’s Modified Dulbecco’s Medium (13390, Sigma-Aldrich) supplemented with 20% fetal bovine serum (CH30160,03, Hyclone) and 1%L-glutamine (G7513, Sigma-Aldrich).

### Proton release measurements

A cytosensor^®^ microphysiometer (Molecular Devices, San Diego) was used to analyze the metabolism of human lymphocytes as described elsewhere^9^,^10^. In brief, the device harbors a pH–sensitive silicon biosensor which measures changes in the current–voltage relation when protons are released into the bath solution. The charged groups originate from a thin insulating layer of silicon oxide and nitride. The measuring compartment of the micro–chamber containing the cells had a volume of 1.4 μl and was perfused discontinuously with a weakly buffered culture medium at a rate of 50 μl/min and a temperature of 37 °C.

**Figure 1:**
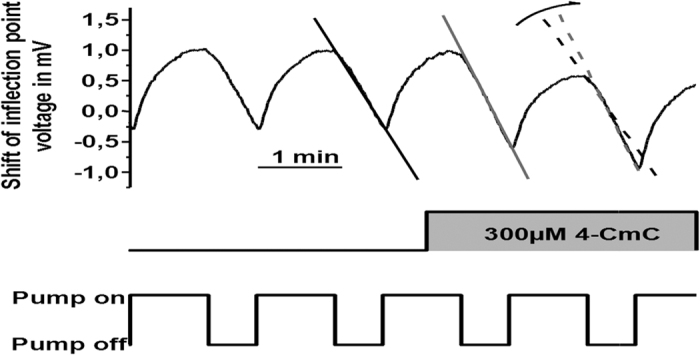
Time–resolved determination of the proton secretion rate from native human blood B–lymphocytes. Four consecutive pump cycles of the perfusion system and corresponding pH–dependent alterations in the change of slope due to the combined action of the proton extrusion from the B–lymphocytes and the flow rate of the perfusion system. The voltage within the chamber represents a measurement of the proton concentration. During the intervals labelled “pump–on”, the chamber was perfused with experimental solution. The “pump–off” intervals prevent the removal of protons, resulting in a change of the inflection points of the tension line. During this interval, the acidification rate was determined from the pH decrease.

Figure 1 shows four consecutive perfusion cycles during which the proton concentration in the chamber equilibrated with that of the bath solution. Cessation of perfusion (i.e. ‘no flow’ interval) results in the accumulation of extruded protons from cells within the chamber (i.e. decreased pH), which invokes a reduction in the potential difference or decreased voltage at the biosensor. To determine the acidification rate corresponding to the metabolic activation of B–lymphocytes, simple linear regression was used to determine the slope of a 30 second interval of the voltage–time curve. The chambers were perfused for a minimum of seven minutes between consecutive additions of trigger substances to ensure equilibration. The amplitude of recorded signals was typically in the order of 0.01–0.1 pH–units/min. Thus, the timing of the perfusion cycle reflected a compromise between the temporal resolution of the measurement and the time required to determine the acidification rate during the ‘no–flow’ interval.

**Figure 2:**
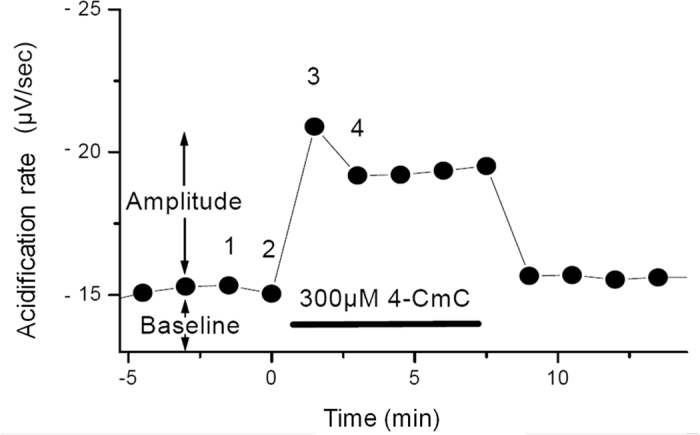
Acidification rates, as determined in Fig. 1, plotted versus time. Numbers 1 to 4 indicate the four perfusion cycles shown in B. 4–CmC = 4–chloro–m–cresol.

Figure 2 shows the time–resolved change in acidification rate of isolated B–lymphocytes caused by addition of 300 μM 4–CmC. Weakly buffered culture medium was pumped discontinuously through the sample volume. Each measurement point corresponded to a single pump cycle of the perfusion system. In the ‘no flow’ interval, the pH decreased due to cellular proton release. Any stimulus that activated cell metabolism e.g. application of 300 μM 4–CmC, resulted in a further change in the slope of the curve, which represented the acidification rate. Control of the pump cycle, data acquisition and acidification rate calculation were carried out with the system’s microcomputer (Macintosh Power PC 7600/132) and proprietary software (Cytosoft^®^ program, Molecular Devices, San Diego).

4–CmC was chosen for microphysiometry as it is a potent activator of RyR1 mediated Ca^2+^ release, and exhibits 15 times higher affinity for RyR1 compared to caffeine^21^. Ryanodine was chosen as a second application substance for evaluation of the microphysiometry method.

### Statistical Analysis

StatXact (Version 5, Cytel Software, Cambridge, MA) was used for all statistical procedures. Shapiro Wilkes tests were used to evaluate data for underlying assumptions of normality. Outcome variables were determined to be normally distributed, so means and standard deviations have been used as summary statistics. Potential differences between groups in acidification rates were evaluated using Wilcoxon Mann Whitney test. An alpha level of 0.05 was used for all tests of significance.

## Results

### Characterization of individuals

In total, 24 individuals underwent IVCT during the two year study period. According to the European IVCT protocol, thirteen were classified as MHS and seven as MHN. Four additional patients served as healthy controls. All individuals were genetically independent and not related. Genetic screening identified that ten of the thirteen individual’s with MHS possessed a causative RyR1 mutation. Native B-lymphocytes of nine MHS, five MHN and four controls were exposed to 4-CmC to measure acidification rate. Within those, a Gly–2434–Arg mutation was detected in five patients and an Arg–614–Cys mutation in one other (Table 1). To increase test specifity, immortalized B-lymphocytes of three further MHS and two MHN individuals were exposed to ryanodine. Within those three different mutations were detected: Cys4664Arg, Arg530His, Arg2163Pro. Age distribution, gender balance and body mass index (BMI) were comparable between groups. The mean (SD) age in the MHS group was 34.6 (17.0) years and 29.2 (10.7) years in the MHN group, while the mean BMI was 23.6 (2.9) and 23.7 (2.4) years in the MHS and MHN groups, respectively. The presence of increased CK–levels in MHS patients is well known; average CK–levels in MHS was 63 U/L compared with 38 U/L in MHN patients. Histological investigation detected no specific pathological changes and was unable to differentiate between groups.

### B–Lymphocyte separation

B–lymphocytes were isolated from 18 ml samples of whole blood. On average 23.6 ± 10.0 million leukocytes were counted after the density gradient centrifugation using a Neubauer counting chamber. This equates to a cell count of 1311 ± 555 leukocytes/μL of investigated whole blood, which when compared to the published values (450–10100 leukocytes/μL) was in the lower range of normal^22^. On average, 14.7 ± 8.0 million leukocytes were detected after temporary cryopreservation. After negative isolation, 1.8 ± 1.2 million B–lymphocytes (0.10 ± 0.07 million per μl extant), were transfected in low melt agarose (1.5%) cups for measurement of proton release. The number of B–lymphocytes did not differ significantly between groups.B–lymphocytes per μl of whole blood were 0.10 ± 0.07 in the MHS group, 0.10 ± 0.05 in the MHN group (Table 2).

### 4–CmC induced acidification responses in normal and MHS native B–lymphocytes

**Figure 3:**
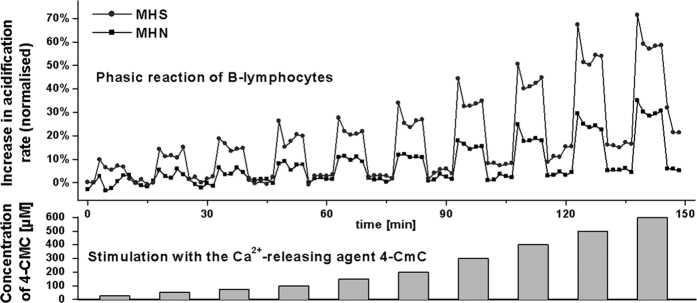
Original traces of acidification responses of native B–lymphocytes at increasing 4–chloro–m–cresol concentrations. Filled circles show the normalized increase in proton secretion rate recorded from a malignant hyperthermia susceptible (MHS) patient confirmed by IVCT. The patient was a carrier of the Gly–2434–Arg mutation. Filled squares depict rate recordings from a malignant hyperthermia–negative (MHN) patient confirmed by IVCT.

Figure 3 demonstrates the acidification–rate responses of B–lymphocytes from a single MHS and MHN patient. The concentration of 4–CmC was gradually increased from a minimum value of 25 μM to 600 μM. Each drug application consisted of a seven minute exposure with the respective concentration and a comparable washout period with 4–CmC–free solution. Ordinate of the upper panel shows the change in acidification rate as a percentage of the initial (predrug) basal rate. The responses were qualitatively similar, in that a step change in 4–CmC concentration resulted in a rapid, though transient, increase in acidification rate. The rate remained elevated above baseline during the time of stimulus, although at a lower level than the transient peak, and only decreased below baseline after return of the control solution.

**Figure 4:**
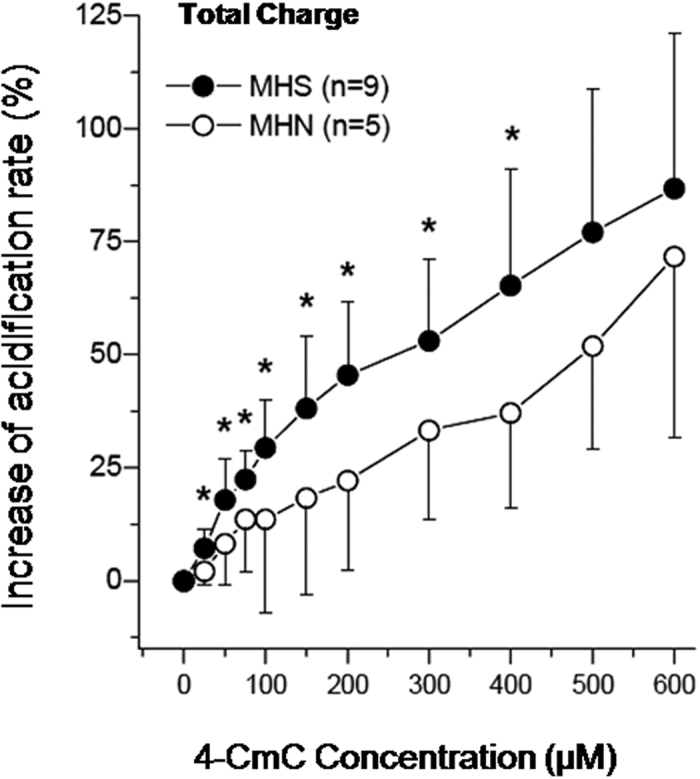
Dose dependence of the acidification rate in IVCT–confirmed malignant hyperthermia–negative (MHN). Susceptible (MHS) human B–lymphocytes. Increase in acidification rates (percent of pre–drug value) plotted as a function of 4–chloro–m–cresol (4–CmC) concentration. Total normalized increase in acidification rate. Mean data from nine MHS patients (filled circles) and five MHN (open circles). Error bars show SD; asterisks indicate p < 0.05 using the exact Wilcoxon rank sum test.

**Figure 5:**
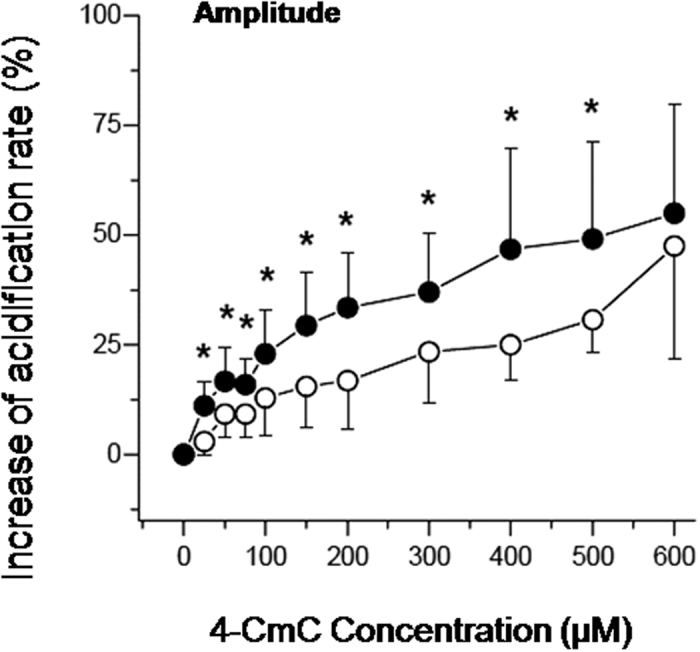
Normalized peak amplitude. Mean data from nine MHS patients (filled circles) and five MHN (open circles). Error bars show SD; asterisks indicate p < 0.05 using the exact Wilcoxon rank sum test.

**Figure 6:**
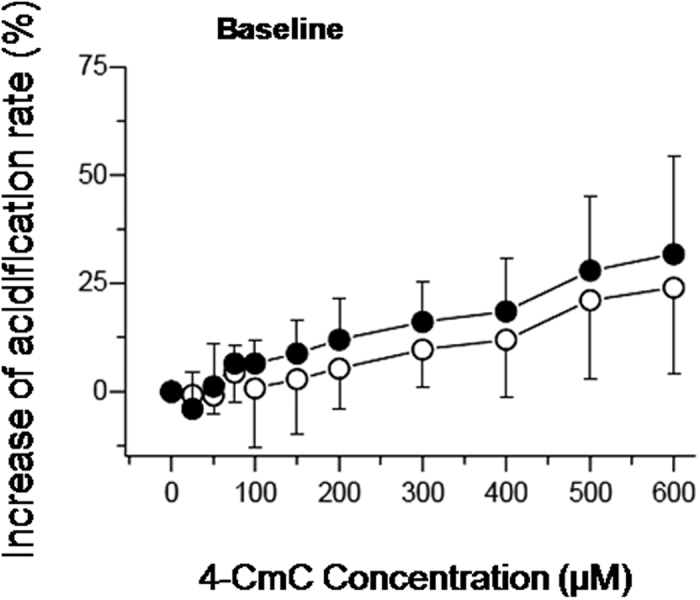
Normalized increase in baseline acidification rate. Mean data from nine MHS patients (filled circles) and five MHN (open circles). Error bars show SD; asterisks indicate p < 0.05 using the exact Wilcoxon rank sum test.

Figure 4 illustrates the total change in acidification rate of MHS and MHN patients. In Fig. 5 the amplitude of the transient response on application of 4–CmC is shown. Figure 6 shows the change in baseline rate, measured immediately before stimulation with 4–CmC. The total change in acidification rate, which comprises the amplitude of both the transient increase and baseline, was significantly increased in the MHS group compared to the MHN group (15.0% vs. 6.7%, p < 0.05). On average, stimulation of MHS lymphocytes with 4–CmC over a range of concentrations (50–400 μM) resulted in a magnitude shift of the total dose-response curve, which is in the order of a twofold increase as compared to MHN reactions. (p < 0.05). In the MHN group, B–lymphocytes needed 2–3 times higher concentrations of 4–CmC to reach a similar increase in total acidification rate as MHS. Baseline values, however, were not significantly different between groups.

### Comparison of IVCT and microphysiometer test results of B–lymphocytes

With IVCT, a contracture force of 2 mN during exposure to a certain concentration of trigger agent (halothane or caffeine) is considered diagnostic for MHS. All biopsies used for this investigation had shown unambiguous results in IVCT, i.e., concentration thresholds for contractures of muscle fiber bundles triggered by halothane or caffeine were either normal (MHN, n = 5) or clearly shifted to lower concentrations (MHS, n = 9). In addition, we also measured 4–CmC–induced force responses in 11 of 14 muscle biopsies. At concentrations of 25 μM, 4–CmC resulted in contracture forces of 2 mN or above in previously determined MHS positive biopsies (halothane–caffeine challenge). MHN biopsies, however, only reached comparable contracture force at concentrations above 150 μM.

**Figure 7:**
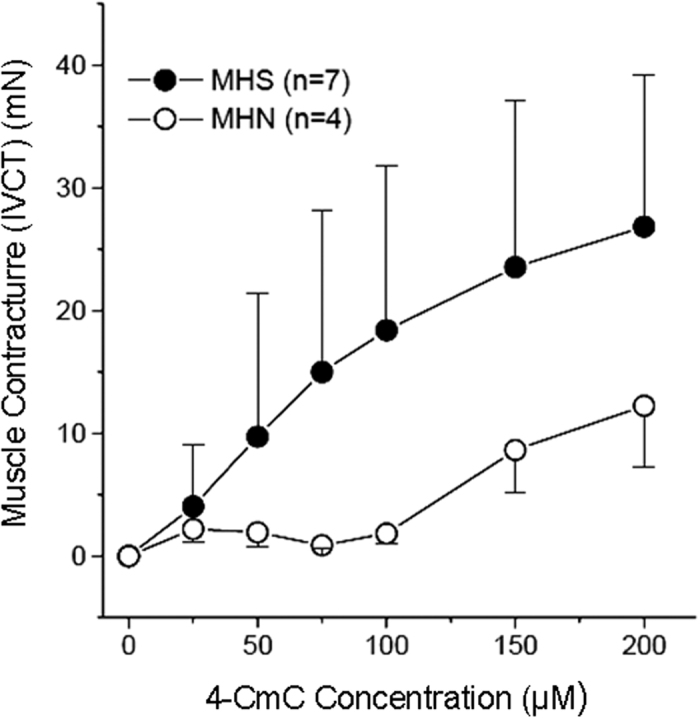
Comparison of contracture tests and acidification assay. Contracture force from the *in vitro* contracture test plotted relative to 4–chloro–m–cresol (4–CmC) concentration. Error bars indicate SD.

**Figure 8:**
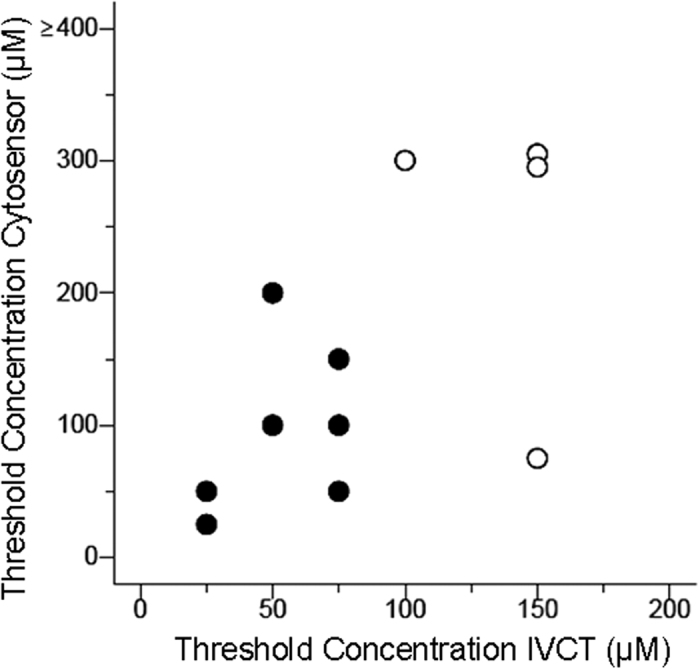
Scatter plot of IVCT data and microphysiometer data at given 4–CmC concentrations reflecting a supra–threshold drug response (threshold definitions, 25% increase above baseline value of acidification rate in the Cytosensor test [ordinate] and 2 mN concentrations force in the IVCT [abscissa]).

Comparison between individuals revealed a positive association between the strength of the 4-CmC induced IVCT response of muscle and the acidification assay of B–lymphocytes (Figs 7 and 8). There was insufficient data, however, to calculate a robust receiver operator characteristic curve to compare tests. Consequently, we estimated the sensitivity and specificity of the test using an arbitrary cut-off point. The cut-off point was selected under the presumption that maximum sensitivity rather than specificity is critical in MHS. Based on our data, we considered a total acidification rate of >25% [at 200 μM 4-CmC] above baseline as a pathological threshold. Using this cut-off point, the assay had a sensitivity of 100% and a specificity of 70%.

### Ryanodine induced acidification responses in normal and MHS immortalized B-lymphocytes

**Figure 9:**
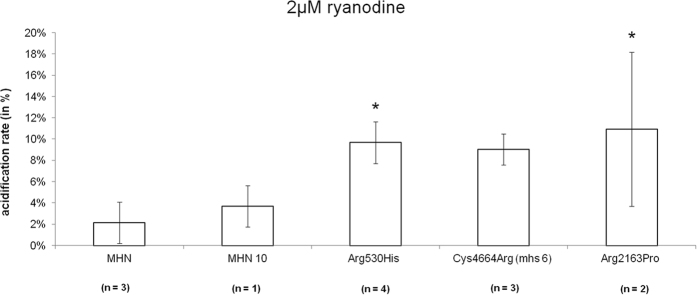
Effect of 2 μM ryanodine on acidification rate in immortalized B-lymphocytes of two MHN and three MHS individuals. Multiple measurements (n) for each individual were included. Mutants Arg2163pro and Arg530His were significantly (p < 0.05) different form MHN individuals. Error bars show SEM.

Figure 9 demonstrates the acidification-rate responses of immortalized B-lymphocytes from four MHS and two MHN patients. The dose of 2 μM ryanodine resulted in a less pronounced increase in acidification rate in MHS compared to MHN individuals.

## Discussion

We have shown previously that the use of a highly sensitive proton biosensor assay, can identify MHS by measuring the cellular acidification rate of both cultivated myotubes and EBV-immortalized lymphoblastoid cell lines^9^,^10^. In the current study, we investigated whether evaluating metabolic activity in native human B–lymphocytes might offer a future minimally–invasive approach for MH–diagnostics. For each test, B-lymphocyte separation took approximately 25 minutes and measurement of the 4-CmC induced acidification rate required, on average, only 160 minutes. To apply microphysiometry to human blood cells, we recorded extracellular acidification of human B–lymphocytes with a sensitive pH–metric biosensor. The acidification rate that occur after exposure to each test chemical concentration are calculated and compared to the mean basal acidification rate of the same cells prior to exposure to the test chemical. Stimulation of RyR1 causes intracellular Ca^2+^ mobilization, resulting in an increased energy demand, i.e. ATP, which is re–synthesized by the respiratory chain and glycolysis^22^. The main products of this reaction are CO_2_ and lactic acid, which results in proton secretion from the cell. Similar to muscle cells, stimulation of B–lymphocytes caused a phasic and dose-dependent increase in acidification rate^9^. The reaction sequence shows an initial transient peak in acidification rate followed by an elevated plateau phase for the duration of stimulation. This is a common signal characteristic of acidification measurements after receptor stimulation^23^. A decrease of intracellular pH level results in the activation of the Na+/H– exchanger, which in turn causes the transient spike within the measurement signal. The subsequent plateau is likely due to a negative feedback mechanism of the cell. An increase in Ca^2+^ in the millimolar range causes an inactivation of RyR1 and reuptake of Ca^2+^ in the sarcoplasmic reticulum^24^. Moreover, rapid ATP consumption, which is necessary for complete RyR1 activation, seems to be a contributing feature^24^. Due to the limited temporal resolution of the measuring system and the rapid kinetics of Ca^2+^ secretion with 4–CmC stimulation, it is likely that peak acidification levels of B–lymphocytes were underestimated by the system used in the current study^25^.

To the best of our knowledge, this is the first study to reveal the potential of evaluating the risk of MH-disposition in human individuals using peripheral blood and independent of underlying RyR1 genetic mutation. Caused by the genetic heterogeneity, a metabolic assay is indispensable for the diagnosis of MH-disposition. In this study we advanced previous work of our group showing that MHS myotubes show an abnormal proton metabolism and corroborate the recent findings of Bina *et al.* demonstrating an increased accumulation of adenosine in lymphocytes from MHS patients^26^. In this study, both the total and peak acidification rate of native B–lymphocytes was higher in MHS than MHN patients. Differences between MHS and MHN patients in acidification rates were significant at 4–CmC concentrations between 50 and 400 μM. The greatest difference in acidification rate between groups was detected at a 4–CmC concentration of 200 μM. Higher concentrations caused a nearly parallel increase in total acidification rate in both MHS and MHN groups (Fig. 3). Although further research is required, this effect might have been due to nonspecific activation of further Ca^2+^ signaling cascades, such as IP3 or the mitochondrial matrix Ca^2+^ pool^17^. Previous research has suggested that the IP3 cascade system in blood samples was unable to identify MH susceptibility, however recent data suggests that IP_3_R–mediated Ca^2+^ release might contribute to increased intracellular Ca^2+^ levels via additional release^27^. Moreover, the mitochondrial matrix comprises a subcellular Ca^2+^ store which might contribute to accumulation under conditions of de–energization^28^,^29^. Compared to earlier investigations of acidification rates in cultivated muscle cells, B–lymphocytes showed a lower acidification rate at rest and a 5-fold lower secretion reaction after 4–CmC stimulation^9^. This might reflect the lower expression of the RyR1 within the intracellular storage of B–lymphocytes, a lack of the interaction of DHP–R/RyR or further Ca^2+^ signaling cascades^8^. Interestingly, the 4–CmC induced increase in Ca^2+^ in B–lymphocytes of MHS and MHN were compared using flow cytometry^30^. Only concentrations of 4–CmC above 400 μM resulted in significant differences in calcium levels^30^. Early findings dating back to 1987 tentatively suggested altered immune cell function in MH. In due course, flow cytometry showed distinct aberrations in the response of MH-lymphocytes to 4-CmC^31^. The acidification assay used in the current study requires 2 to 3 fold lower 4-CmC concentrations for risk evaluation of MH-disposition.

IVCT has been shown to have a high sensitivity (97–99%) and acceptable specificity (approximately 70%) which may be increased to 94% by using two trigger substances (halothane and caffeine)^4^. The standard protocol of CHCT and IVCT differ in the following points: Most important, IVCT utilizes incremental exposure to halothane and requires only investigation of two muscle bundles for each test substance, CHCT utilizes 3% halothane and requires three muscle bundles for each test substance^32^. CHCT was reported to have a sensitivity of 97% and a specificity of 78%^33^. However, evaluation of muscle biopsies with IVCT has been shown by some to have high variability. For instance, poor agreement has been reported in 12% of cases when a standardized IVCT approach was used by two MH centers to evaluate muscle biopsies from the same patients^32^. While the halothane test was shown to be more accurate and reproducible than the caffeine test, additional application of 4–CmC as a stimulant has been shown to increase the specificity of the IVCT test^34^,^35^. In a set of exploratory experiments, the effects of halothane were investigated in the microphysiometry set up. However, the results were inconsistent, which we attributed to technical difficulties associated with the use of silicon componentry and the volatile nature of the drug. To potentially increase test specifity, we investigated ryanodine as another test substance. The ryanodine-induced acidification increase, however, was less prounced compared to 4-CmC even though multiple testing of B-lymphocytes was performed after immortalization. Similarly, ryanodine testing was approved as an additional test agent in IVCT/CHCT, but showed an overlap between MHS and MHN groups^36^. Although the ryanodine contracture test may help diagnose susceptibility to malignant hyperthermia the results showed high variability, which might be at least in part be explained by the pharmacological nature of exclusive “open state” receptor binding^36^.

Metabolic activity of muscle is influenced by several individual factors including nutrition, pre–existing disease, inflammation and hormonal levels e.g. higher thyroxine, cortisol and insulin levels have been detected in MHS positive individuals which might impact IVCT test results. However, microphysiometry (acidification) is also an indirect measure of Ca^2+^ release. Therefore, disorders that affect Ca^2+^ turnover, energy metabolism and proton handling have the potential to adversely influence acidification rates determined via this method. Moreover, microphysiometry of B–lymphocytes depends on the function of isolated B–lymphocytes from the peripheral blood sample. The CD19 + B-cells comprise a heterogeneous cell population which in turn is influenced by a number of physiological and pathophysiological conditions. Nonetheless, the findings of the current study suggest that microphysiometry of B-lymphocytes may be a useful first line adjunct for the evaluation of MH susceptibility.

Mutations of RyR1 or CACNA1S are only detectable in about 50% to 70% of the MH families^37^. Moreover, alternative pathomechanisms, such as changes of cortisol and thyroxine levels or membrane lipid composition, are not detected using standard genetic approaches^38^. Accordingly, genetic screening by DHPLC identified only mutations in six of nine MHS individuals diagnosed by IVCT and acidification measurements. Since not all amplicons are sequenced with the same efficiency, some may be underrepresented or missed completely resulting in undetected variants^37^. Recently, exome sequencing revealed rare variants of RyR1 and CACNA1S which were excluded before using Sanger sequencing^39^. In three of our nine MHS individuals a mutation was not detected by a follow up investigation using Sanger sequencing. Until cost and time-effective next generation sequencing is developed for whole genome analysis, the functional approach seems to be a more reliable approach for MHS screening^40^,^41^. With 106 exons, to date only 34 *RYR1* mutations have been described as causative for MH and/or central core disease^42^,^43^,^44^,^45^. For the other roughly 400 known RYR1 variants, functional data are still necessary for the proof of pathogenicity. At this point a metabolic assay might offer a future perspective. Most notably, the required sampling may be processed decentralized. Detection of non RyR1 mediated forms of MH via a metabolic assay of lymphocytes, however, remains uncertain. Microphysiometry of B-lymphocytes, as applied in the current study, needs to be studied more extensively with multiple underlying genetic variants before it could be clinically used as a pre-screening test for MH.

## Conclusion

While further research is required, the findings of our study, highlight the potential of metabolic assays of native B–lymphocytes from peripheral blood to be used as a pre–screening tool for MHS or as an adjunct to IVCT. Native B–lymphocytes from MHS individuals are more sensitive to 4–CmC than those from MHN, reflecting a greater Ca^2+^ turnover. The acidification response, however, was less pronounced than in muscle cells, presumably reflecting the lower expression of RyR1 in B–lymphocytes. Metabolic assays of B–lymphocytes might be a viable but less–invasive adjunct to IVCT for pre–screening in MHS susceptibility. The sensitivity and specificity of the 4-CmC increased acidification rate resembles the range of the IVCT using only one test substance. Additional research is required to identify additional test substances for microphysiometry and to further elucidate the sensitivity and specificity of the technique prior to potential clinical use. Nonetheless, this study provides the first evidence that a functional test approach using 20 ml human blood may discriminate between MHS and MHN individuals.

## Additional Information

**How to cite this article**: Hoppe, K. *et al.* Hypermetabolism in B–lymphocytes from malignant hyperthermia susceptible individuals. *Sci. Rep.* **6**, 33372; doi: 10.1038/srep33372 (2016).

## Figures and Tables

**Table 1:** Table represents the IVCT with halothane and caffeine and the 4-CmC Acidification threshold form MHS and MHN individuals.

	Mutation	Halothan		Coffeine		4-CMC		
		Threshold (mM)	Contraction (mN)	Threshold (mM)	Contraction (mN)	Threshold (μM)	Contraction (mN)	Acidification threshold >25% (μM)
MHS 1	Gly-2434-Arg	0,22	3	2	2	50	5	100
MHS 2	Gly-2434-Arg	0,22	2,2	1,5	4,3	not determined	not determined	75
MHS 3	Gly-2434-Arg	0,22	4	1.5	3	not determined	not determined	150
MHS 4	Arg-614-Cys	0,44	2,6	2	2,5	50	5,5	200
MHS 5	none detected	0,44	2,4	2	2,4	75	3,2	100
MHS 6	Gly-2434-Arg	0,22	2	1	3,8	25	9	25
MHS 7	Gly-2434-Arg	0,44	3,1	1	5,6	75	5,4	50
MHS 8	none detected	0,44	7	2	7	75	2,5	150
MHS 9	none detected	0,22	3,4	1	4,9	25	14,2	50
MHN 1	none	>0,88	1,2	3	1.9	not determined	not determined	300
MHN 2	none	>0,88	1,6	32	1	150	0,8	75
MHN 3	none	>0,88	0,2	4	−1,4	150	−1,4	300
MHN 4	none	>0,88	0,6	4	0	150	0,8	300
MHN 5	none	>0,88	0,9	3	−1,6	100	1	300

A Gly-2434-Arg mutation was identified in the MHS individual 1, 2, 3, 6 and 9, an Arg-614-Cys mutation in MHS 4. In MHS 5, 7 and 8 neither a RyR1 mutation nor a CACNA mutation was identified.

**Table 2:** Table represents total amount of isolated Lymphocytes in the MHS, MHN and healthy controls.

MHS	Leukocytes prae Cryo-preservation (in mio)	Leukocytes after Cryo-preservation (in mio)	Leukocytes prae Cryo-preservation (in %)	B-Lymphocytes (in mio)	B-lymphocytes (per ml whole blood)
MHS 1	17.8	11.1	64.0	2.7	0.15
MHS 2	19.5	10.5	54.0	1.9	0.11
MHS 3	18.8	11.1	59.0	0.9	0.05
MHS 4	23.3	13.5	58.0	4.5	0.25
MHS 5	27.4	17.3	63.0	2.7	0.15
MHS 6	9.5	5.0	53.0	0.6	0.03
MHS 7	6.2	3.8	61.0	0.4	0.02
MHS 8	30.0	22.3	74.0	2.66	0.015
MHS 9	0.8	0.4	50.0	0.16	0.001
	17.0 ± 9.2	10.5 ± 6.4	60.0 ± 6.8	1.8 ± 1.4	0.1 ± 0.7
MHN					
MHN 1	23.0	6.5	28.0	0.66	0.04
MHN 2	21.1	9.4	44.0	1.8	0.1
MHN 3	26.0	17.9	68.0	1.16	0.06
MHN 4	37.0	24.0	64.0	3.1	0.17
MHN 5	36.0	21.0	58.0	2.0	0.1
	28.6 ± 6.6	15.8 ± 6.7	52.0 ± 14.6	1.7 ± 0.8	0.09 ± 0.04
Control					
C 1	38.0	32.4	85,0	3.3	0.18
C 2	28.5	14.8	52,0	0.72	0.04
C 3	30.9	17.5	57,0	3.0	0.17
C4	28.8	23.3	81,0	0.6	0.03
	31.6 ± 3.8	22.0 ± 6.7	69.0 ± 14.4	1.9 ± 1.3	0.11 ± 0.07
